# Sleep in Children With Pallister Killian Syndrome: A Prospective Clinical and Videopolysomnographic Study

**DOI:** 10.3389/fneur.2021.796828

**Published:** 2021-12-16

**Authors:** Anna Fetta, Veronica Di Pisa, Martina Ruscelli, Luca Soliani, Giacomo Sperti, Sara Ubertiello, Emilia Ricci, Greta Mainieri, Alessandro Rocca, Maria Margherita Mancardi, Lucio Giordano, Dario Pruna, Aglaia Vignoli, Federica Provini, Duccio Maria Cordelli

**Affiliations:** ^1^IRCCS Istituto delle Scienze Neurologiche di Bologna, UOC di Neuropsichiatria dell'Età Pediatrica, Bologna, Italy; ^2^Dipartimento di Scienze Mediche e Chirurgiche (DIMEC), Università di Bologna, Bologna, Italy; ^3^Scuola di Specializzazione in Pediatria - Alma Mater Studiorum, Università di Bologna, Bologna, Italy; ^4^Child Neuropsychiatry Unit, Department of Health Sciences, Epilepsy Center, San Paolo Hospital, University of Milan, Milan, Italy; ^5^Dipartimento di Scienze Biomediche e Neuromotorie (DIBINEM), Università di Bologna, Bologna, Italy; ^6^Department of Medical and Surgical Sciences (DIMEC), Pediatric Emergency Unit, IRCCS Sant'Orsola Hospital, Bologna, Italy; ^7^Child Neuropsychiatry Unit, Department of Medical and Surgical Neurosciences and Rehabilitation, IRCCS Istituto Giannina Gaslini, Genova, Italy; ^8^Child Neuropsychiatric Division, Spedali Civili, Brescia, Italy; ^9^Department of Pediatric Neurology and Epileptology, Pediatric, ARNAS Brotzu, Cagliari, Italy; ^10^Child Neuropsychiatry Unit, Department of Health Sciences, ASST Grande Ospedale Metropolitano Niguarda, University of Milan, Milan, Italy; ^11^IRCCS Istituto delle Scienze Neurologiche di Bologna, Bologna, Italy

**Keywords:** PKS, SDSC, video polysomnography, sleep disorders, rare disease

## Abstract

**Objectives:** Pallister-Killian syndrome (PKS) is a rare genetic disorder with multi-organ involvement caused by mosaic tetrasomy of chromosome 12p. Although many caregivers report the presence of impaired sleep in their children, there are no clear data in the literature on this issue and no systematic study has ever been performed. With this study, we aimed to characterize the features of sleep in Pallister-Killian syndrome and identify the possible influence of clinical and demographic features. Moreover, our aim was to verify the effectiveness of conventional screening questionnaires in this particular group of patients.

**Methods:** We prospectively enrolled 14 patients aged 1–17 years in collaboration with PKS Kids Italia ONLUS. The Sleep Disturbance Scale for Children (SDSC) questionnaire was administered to caregivers. Then, video polysomnography (VPSG) of at least 24 h was performed and results were compared with a same-aged control group.

**Results:** A total of 92% of patients had abnormal SDSC scores, extremely high in the “disorder of initiating and maintaining sleep” (DIMS) and “sleep breathing disorders” (SBD) subscales. VPSG showed a significantly impaired macrostructure in PKS patients, with a higher Arousal Index (*p* < 0.00001) and percentage of time spent in N3 (*p* < 0.00001), and reduced Sleep Efficiency (*p* = 0.0006). After dividing both PKS and controls into two groups based on median age, some peculiarities emerged: the younger group had higher Awakenings Index (*p* = 0.0207) and percentage of time spent in N1 (*p* = 0.015) while the older group showed higher time in bed (TIB) (*p* = 0.0485), compared with controls. Due to poor compliance, the Apnea-Hypopnea Index (AHI) was evaluated only for 10 PKS children, being significantly increased (*p* = 0.0427) compared with controls. SBD subscale scores in SDSC were significantly related to AHI values in VPSG (*p* = 0.0099).

**Conclusions:** This study constitutes the first attempt to describe the sleep pattern in PKS. Despite small numbers due to the rarity of the syndrome, our VPSG results confirm the high prevalence of sleep disorders (SDs) in these patients. It is therefore essential to investigate and treat them. The SDSC scale is a good screening tool for early detection also in these patients, with particular sensitivity in detecting breathing disorders.

## Introduction

Pallister-Killian syndrome (PKS) or 12p tetrasomy is a tissue-limited mosaic aneuploidy, in which most cases are due to the presence of a supernumerary 12p isochromosome (1). The phenotypic spectrum is broad, characterized by facial dimorphism, skin pigmentation differences (hypopigmentation/hyperpigmentation), multiple congenital anomalies (including cerebral malformation), hypotonia, epilepsy, neuromotor delay, and intellectual disability (ID) (2). Although the phenotype is variable, most of the patients have a severe neurological impairment that highly impacts on their life (3).

Sleep is frequently impaired in children with neurodevelopmental disorders (NDDs) and multiple disabilities (4). Moreover, differently from typically developing (TD) children, the ones in NDDs tend to be chronic, lasting into adolescence or adulthood (5).

A recent meta-analysis investigated the prevalence of sleep disorders (SDs) in 19 rare genetic syndromes (GSs) and found an average higher frequency than in TD children, with extreme variability in the type of sleep disorder across syndromes (6).

Several anatomical, physiological, and neurological factors, typical for the different GSs, may contribute to altering sleep: the altered melatonin secretion leads to circadian rhythm disruption in Smith–Magenis syndrome, while hypotonia determines frequent breathing disorders in Down and Angelman syndrome (7, 8). Other frequent causes are poor sleep hygiene, pain caused by co-occurring medical conditions, epilepsy, and adverse effects of drug therapies (6, 9). It is important to correctly investigate the pattern of alteration to assess the most appropriate treatment (5).

The altered sleep quality may further lead to increased daytime drowsiness, restlessness, and may even exacerbate the underlying disease (i.e., lower seizure threshold in epileptic disorders, increased level of distress, or impaired regenerative processes). These processes create a vicious cycle that worsens SDs with consequences on the quality of life of the entire household (4).

SDs seem to be a frequent issue also among individuals with Pallister-Killian syndrome and caregivers often report them. Apneic episodes have been occasionally described (10). Kostanecka et al. in 2012 evaluated 15 children with the Aberrant Behavior Checklist (ABC) finding a prevalence of sleepiness and lethargy during daytime activities; nocturnal sleep problems were reported only in 5 children (4 reports of waking up at night and 1 of sleep apnea) (11). Up to now, no systematic direct instrumental observation has been performed.

With this study, we aimed to characterize the sleep in PKS children through video-polysomnographic (VPSG) analysis and identify the possible influence of clinical and demographic features. Moreover, our aim was to verify the reliability of SDSC, as a first screening tool, in recognizing the main SDs in this particular group of individuals.

## Materials and Methods

### Participants

#### Study Population

Fourteen individuals with genetically confirmed PKS (nine females and five males, aged 12 months-17 years and 4 months) were recruited in collaboration with the “PKS Kids Italia ONLUS” association. Clinical-anamnestic data were collected.

Secondly, children were sorted on the basis of age and then split into two subgroups of 7 individuals each: subgroup A (5F/2M; aged 12 months to 7 years and 3 months), and subgroup B (4F/3M; aged 11 years and 6 months to 17 years and 4 months).

#### Control Group

The Control group was composed of 14 individuals aged 1–16 years (8F/6M) with no history of neurological disorders, ID, and SDs.

Similarly to the PKS population, 2 control subgroups were identified: subgroup Ac (4F/3M aged 1–10 years) and subgroup Bc (4F/3M aged 11–16 years). Those subgroups were then paired, respectively, with subgroup A and subgroup B for the age-dependent analysis.

### Tools

#### Screening Questionnaire

As a first step, the SDSC questionnaire was administered to the caregivers of PKS children. SDSC consists of 26 items in a Likert-type scale with values 1–5 (1-never; 2-occasionally; 3-sometimes; 4-often; 5-always) (12, 13). The scale comprises a total score and six subscales: disorders of initiating and maintaining sleep (DIMS), sleep breathing disorders (SBD), disorders of arousal (DA), sleep-wake transition disorders (SWTD), disorders of excessive daytime somnolence (DOES), and sleep hyperhidrosis (SHY). Scores are then normalized on a scale of 1–100 and categorized as follows: normal (under 50), borderline (between 50 and 70) or clinical disorder (above 70).

#### Video-Polysomnography

Subsequently, all patients underwent a VPSG lasting at least 24 h in our sleep laboratory.

Recordings comprised 19 EEG channels, left and right electrooculogram (EOG), the chin electromyography, electrocardiogram, and the respiratory signals (airflow, movements of the chest wall and abdomen, O_2_ saturation of arterial blood).

Sleep was staged and scored according to the American Academy of Sleep Medicine (AASM) 2017 criteria (14) by two of the investigators (VD and SU). The following parameters were evaluated:

- time in bed (TIB): time between light-off and light-on
- sleep period time (SPT): minutes from sleep onset to the end of the final sleep epoch
- total sleep time (TST): minutes from sleep onset to the end of the final sleep epoch minus the time spent awake i.e., total time spent in sleep
- sleep efficiency index (SEI): percentage ratio between total sleep time and time in bed (TST/TIB^*^100)
- sleep onset latency (SOL): minutes from lights out to sleep onset, defined as the first any sleep stage epoch
- first REM latency (FRL): minutes from lights out to first REM
- wakefulness after sleep onset (WASO): time spent awake after sleep onset
- percentage of TST spent in each stage (N1, N2, N3, REM)
- arousal index (AI): number of arousals per hour of TST
- awakenings index (AWI): number of awakenings per hour of TST
- apnea-hypopnea index (AHI): number of apneas and hypopneas per hour of TST.

### Statistical Analysis

Continuous variables are expressed as median with interquartile range. Comparisons between two groups were made using U Mann Whitney test for independent data sets. Correlations between continuous variables were tested with the Pearson correlation coefficient. All the analyses were conducted in SPSS version 23 [SPSS Inc., Chicago, IL, USA], Microsoft Windows version, and *p* ≤ 0.05 from 2-sided tests was considered statistically significant.

### Ethics

Informed consent was obtained from the parents of all patients included in the study. The local ethics committee approved the study.

## Results

### Characteristics of the Population and SDSC

The demographic, clinical, and neuroradiological features of the population, as well as the SDSC results, are described in Table 1.

**Table 1:** Demographic and clinical features, and SDSC results.

**Patient**	**Sex**	**Age**	**Epilepsy**	**AED**	**Sleep disorder treatment**	**DIMS**	**SBD**	**AD**	**SWTD**	**DOES**	**SHY**	**TOTAL**
No. 1	F	1y	Yes	No therapy	–	41	51	47	50	46	51	45
No. 2	F	1y	No	–	–	60	45	47	75	42	69	62
No. 3	F	1y, 5m	No	–	–	64	51	47	66	64	45	64
No. 4	M	1y, 5m	No	–	–	73	86	47	41	64	64	70
No. 5	M	2y, 4m	Yes	VGB, VPA	Melatonin	79	100	70	84	81	69	99
No. 6	F	6y	No	–	Melatonin	66	100	94	100	69	69	100
No. 7	M	7y, 3m	Yes	CBZ	–	70	100	100	75	73	45	90
No. 8	F	11y 6m	Yes	VPA	–	60	45	70	58	88	45	69
No. 9	F	11y, 10m	No	N/A	–	60	45	70	41	50	45	51
No. 10	F	12y, 1m	Yes	CBZ	–	70	86	47	41	53	45	62
No. 11	F	13y, 7m	Yes	VPA, CBZ	–	64	58	58	79	58	45	68
No. 12	M	14y, 4m	No	–	–	66	58	70	58	50	45	62
No. 13	M	16y, 4m	Yes	PB, LEV, VGB, CBZ	–	100	100	70	41	77	69	100
No. 14	M	17y, 4m	Yes	LEV, CBZ	Melatonin	73	86	47	50	42	45	62
MEDIAN (IQR)						66 (11.25)	72 (45.5)	64 (23)	58 (31.75)	61 (22)	45 (22.75)	66 (23)

*AED, anti–epileptic drugs; VPA, valproic acid; VGB, vigabatrin; CBZ, carbamazepine; PB, phenobarbital; LEV, levetiracetam. For all patients, the melatonin dosage ranged from 2 to 5 mg*.

About SDSC scores, 13/14 PKS children were within the clinical disorder range (9 patients with borderline values, 4 in the pathological range).

Males tended to report worse scores than females. This difference reached statistical significance in the DIMS (*p* = 0.019) and SBD (*p* = 0.041) subscales.

No significant differences in scores were found between patients with and without epilepsy or between patients taking or not taking melatonin supplementation.

### Sleep Macrostructure

Table 2 shows the VPSG data of PKS children compared with controls of all ages.

**Table 2:** VPSG data of PKS children compared with controls of all ages.

	**PKS (*n* = 14)**		**Controls (*n* = 14)**		**Mann–Whitney**
	**Median**	**IQR**	**Median**	**IQR**	***p***
TIB (min)	659.50	103.00	563.5	67.5	0.0041^*^
SPT (min)	646.00	115.25	545	62.75	0.008^*^
TST (min)	542.50	141.50	522.5	86.25	0.4286
SEI (%)	81.85	25.27	94.3	4.25	0.0006^*^
SOL (min)	8.40	21.30	8.6	3.375	0.4364
FRL (min)	70.50	62.62	75.5	59.15	0.4443
WASO (%SPT)	13.85	24.05	2.35	4.825	0.0011^*^
N1 (%TST)	7.30	4.35	4.15	2.725	0.007^*^
N2 (%TST)	40.50	11.57	52.8	9.125	0.0005^*^
N3 (%TST)	32.50	9.93	16.2	4.025	<0.00001^*^
REM (%TST)	19.20	6.57	27.25	5.575	0.0004^*^
AI (n/h TST)	12.7	11.35	4.4	2.1125	<0.00001^*^
AWI (n/h SPT)	0.95	0.65	0.81	0.9875	0.1788

*IQR, interquartile range; TIB, time in bed; SPT, sleep period time; TST, total sleep time; SEI, sleep efficiency index; SOL, sleep onset latency; FRL, first REM latency; WASO, wakefulness after sleep onset; AI, arousal index; AWI, awakenings index*.

* *p < 0.05*.

Many of the parameters tested were found to be altered in children with PKS, such as increased AI and WASO, reduced SEI, increased time spent in the N3 stage, and reduced time spent in the N2 stage and the REM stage.

In contrast, sleep latency, REM latency, TST, and awakenings index did not differ significantly from controls.

Medians of the different parameters in PKS children showed no significant differences according to sex, presence of epilepsy, or melatonin supplementation.

#### Analysis by age

Tables 3, 4 show the results of the 2 subgroups grouped by age compared with the corresponding control subgroups.

**Table 3:** VPSG data of subgroup A (1–7 y) compared with control subgroup Ac (1–10 y).

	**PKS sub-A (*n* = 7)**		**Controls sub-Ac (*n* = 7)**		**Mann-Whitney**
	**Median**	**IQR**	**Median**	**IQR**	***p***
TIB (min)	679	148.5	573	36	0.0630
SPT (min)	672	176.5	550	26.5	0.0793
TST (min)	460	135.5	527	57.5	0.2611
SEI (%)	79.9	19.8	95.2	1	0.0025^*^
SOL (min)	9.2	15.45	9.7	2.9	0.1762
FRL (min)	99.5	75.5	63	74.25	0.2843
WASO (%SPT)	19.2	24.55	1.3	1.35	0.0025^*^
N1 (%TST)	10.6	7.1	4	5	0.015^*^
N2 (%TST)	36	8.5	54.9	10	0.0052^*^
N3 (%TST)	27.3	9.5	16.2	2.45	0.0011^*^
REM (%TST)	20.2	10.95	28.1	5.15	0.015^*^
AI (n/h TST)	17.7	10.3	5.5	2.25	0.0011^*^
AWI (n/h SPT)	1	0.9	0.32	0.49	0.0207^*^

*IQR, interquartile range; TIB, time in bed; SPT, sleep period time; TST, total sleep time; SEI, sleep efficiency index; SOL, sleep onset latency; FRL, first REM latency; WASO, wakefulness after sleep onset; AI, arousal index; AWI, awakenings index*.

* *p < 0.05*.

**Table 4:** VPSG data of subgroup B (11–17 y) compared with control subgroup Bc (11–16 y).

	**PKS Sub-B (*n* = 7)**		**Controls Sub-Bc (*n* = 7)**		**Mann-Whitney**
	**Median**	**IQR**	**Median**	**IQR**	***p***
TIB (min)	636	70	526	120.5	0.0485^*^
SPT (min)	633	79.5	514	110	0.7927
TST (min)	545	60.5	476	106	0.2207
SEI (%)	89	15.95	91.1	1.7	0.0207^*^
SOL (min)	7.6	21.05	8.3	4.4	0.4483
FRL (min)	69.5	30.75	76	15.55	0.1251
WASO (%SPT)	9	15.55	6.1	2.75	0.0630
N1 (%TST)	6.4	3.35	4.3	0.85	0.1867
N2 (%TST)	41.8	10.3	52.7	5.05	0.0207^*^
N3 (%TST)	35	10.45	15.2	5.2	0.0011^*^
REM (%TST)	18.2	4.8	26.4	4.9	0.0107^*^
AI (n/h TST)	12.1	5.85	4.08	1.65	0.0011^*^
AWI (n/h SPT)	0.9	0.55	0.86	1.19	0.3264

*IQR, interquartile range; TIB, time in bed; SPT, sleep period time; TST, total sleep time; SEI, sleep efficiency index; SOL, sleep onset latency; FRL, first REM latency; WASO, wakefulness after sleep onset; AI, arousal index; AWI, awakenings index*.

* *p < 0.05*.

Many of the findings found in the analysis of the entire cohort were confirmed.

In contrast to the analyses for the whole group, AWI in the group of the younger PKS children and TIB in the group of the older PKS children appear significantly higher than their controls.

### Respiratory Parameters

Due to the poor compliance, respiratory pattern analysis was only possible for 10 individuals (patients 4, 5, 6, 8, 9, 10, 11, 12, 13, 14).

AHI index in PKS children was statistically higher compared to the relative control group (*p* = 0.0427).

**Figure 1:**
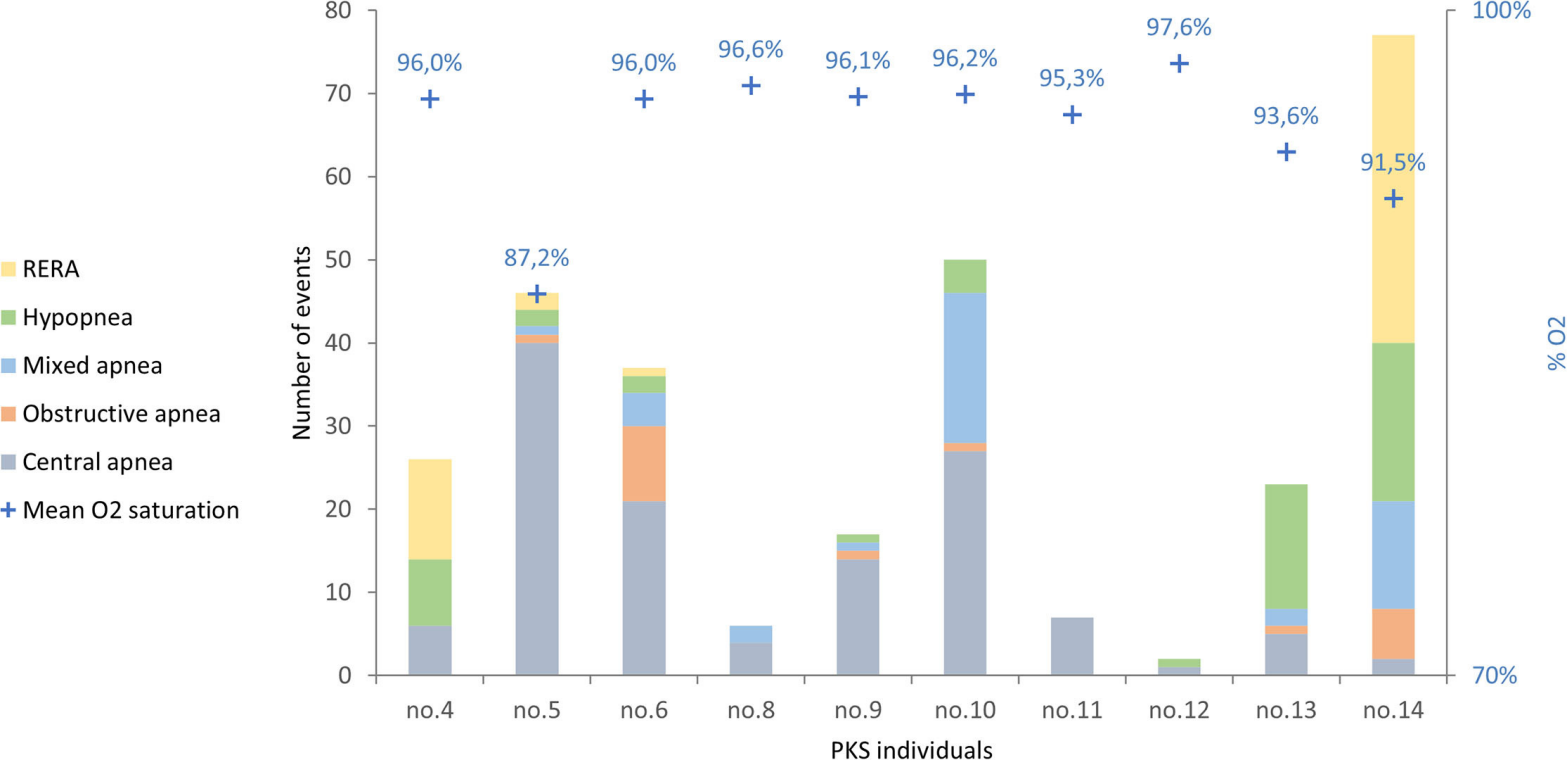
The respiratory events in the 10 PKS subjects evaluated are shown, divided by type of respiratory event. RERA, respiratory effort-related arousal.

The number of respiratory events in our patients ranges between 2 and 77 with an average value of 29 events/h (Figure 1).

The most frequent respiratory events in PKS patients were central apneas registered in 10/10 patients with values ranging from 1/h (patient 12) to 40 apneas/h (patient 5) and an average value of 13/h. Hypopneas were found in 8/10 patients, with an average value of 5/h TST; mixed apneas occurred in 7/10 patients with an average value of 4 apneas/h; obstructive apneas in 6/10 patients with an average value of 2 apneas/h. Moreover, 4 patients presented respiratory effort-related arousal (RERA).

### Paroxysmal Episodes During Sleep

Four patients presented seizures, 5 sleep disorders, and 5 only physiological movements. No patient was observed to have both epileptic seizures in sleep and episodes classifiable as sleep disorders.

As regards the sleep disorders, NREM parasomnias were found in 4: confusional arousals in patients 2, 5, 9, and 10 and night terrors in patient 5. Patient 12 presented an episode of catathrenia (or groaning), classified according to ICSD-3 among respiratory disorders in sleep as isolated symptoms and normal variants.

Among the 4 patients with seizures, one presented generalized myoclonic seizures (patient 1) that were also recorded in wakefulness, one presented exclusively electrical seizures (patient 13), whereas the remaining two patients presented focal seizures (patients 7 and 8).

### Relationship Between SDSC Scores and VPSG Parameters

The total score of the SDSC scale was effective in detecting the presence of SDs, showing borderline or pathological values in 13/14 patients who had abnormal values on VPSG.

**Figure 2:**
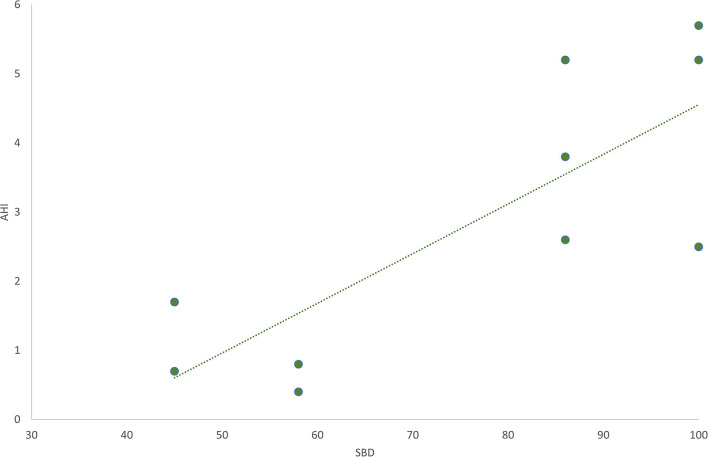
The graphic shows the direct linear correlation between the sleep breathing disorders (SBD) subscale in SDSC test and Apnea-Hypopnea Index (AHI). Pearson's *r* = 0.80; *p* = 0.005.

The SBD subscale was found to be directly correlated with AHI (*r* = 0.80; *p* = 0.005), in the subgroup of patients for whom this parameter was assessed (Figure 2).

## Discussion

This study represents the first attempt to assess sleep features in PKS.

The VPSG allowed us to objectively characterize the sleep pattern, significantly altered in PKS children compared with healthy controls. The highest prevalence of SDs reflects what was previously reported about SDs in the main NDDs (5, 6, 15).

The increased total time spent in bed (TIB), and especially spent sleeping (SPT), can be interpreted as a manifestation of an increased need for sleep, already reported in the study of Kostanecka (11), in which parents reported between 9.5 and 15 h of sleep per day by their children.

Frequent arousals (high AI) and nocturnal awakenings with difficulty falling asleep again (high WASO) determine fragmentation of sleep. As a result of this, sleep efficiency turned out to be significantly impaired (reduced SE).

Regarding sleep macrostructure, significantly increased time spent in the N3 stage and reduced time spent in the N2 stage were found, which however could be due to an overall slowdown in background activity with an overestimation of N3 and consequent underestimation of N2.

Moreover, we found significantly reduced time spent in REM that does not appear to change with growth (as shown by the analysis by age subgroups). This aspect is a frequent finding in GSs and NDDs such as Down syndrome, Smith-Magenis syndrome, Rett syndrome, Angelman syndrome, X-fragile syndrome, and autism spectrum disorder. The causes are likely multifactorial. Certainly, it is known that melatonin is involved in the transition between NREM and REM through its receptors M1 and M2 expressed in different areas that regulate the transition between the two phases (9), thus an alteration at this level could determine an imbalance as in Smith Magenis syndrome (16).

Moreover, neurotransmitter alterations (monoaminergic, glutamatergic, GABAergic, and peptides) could determine an incorrect activation of REM sleep activation mechanisms and/or an easier transition to NREM sleep (5, 17, 18).

REM is fundamental for the development of the CNS in the early stages of life, for memory consolidation, especially sensory and motor, as well as for preparing the CNS for wakefulness (being predominant in the last cycles) (18, 19). For these reasons, a reduced proportion of REM sleep, together with the macrostructure fragmentation, could worsen the characteristic psychomotor development delay of PKS.

The analysis in the two subgroups of patients divided by age confirmed most of the alterations found in the evaluation of the entire PKS group. However, the increased WASO and AWI were observed only in younger patients (subgroup A) and the increased TIB only in older ones (subgroup B). For the latter parameters, therefore, there could be an age-dependent evolution, with a tendency in younger patients to have frequent nocturnal awakenings and lighter sleep, whereas with growth the time spent in bed will increase.

At the evaluation of nocturnal respiratory aspects, apneas and hypopneas were significantly increased in patients with PKS and all presented central apneas, whereas hypopneas, obstructive apneas, mixed apneas, and RERA were observed less frequently.

Central apneas in the pediatric age are most often due to underlying medical conditions, including brain malformations, a common finding among PKS individuals (20). With this regard, further neuroradiological studies could be useful to highlight possible correlations between the different cerebral malformations and these events.

On the other hand, obstructive apneas could be linked to axial hypotonia (as in Down and Angelman syndromes) and typical craniofacial dysmorphisms of PKS, such as macroglossia, micrognathia, and mandibular prognathism, which are known to predispose to upper airway obstruction (3, 10). In this regard, targeted otolaryngological evaluations will be necessary as well as careful pneumological/anesthesiologic follow-up to assess the need for non-invasive mechanical ventilation during sleep.

Respiratory issues, along with altered sleep macrostructure could be at the origin of the lethargic and drowsy attitude during the daytime observed by Kostaneka (11), even if it cannot be excluded that the tendency to sleep more hours than necessary could be a manifestation of a primary altered sleep-wake rhythm. Moreover, non-restorative sleep could directly affect behavioral and emotional aspects, prominent in PKS (15). Finally, it is important to differentiate non-epileptic events from possible seizures during sleep. For all these reasons, potential SDs must be investigated and treated in PKS individuals.

As demonstrated by the characterization of paroxysmal events in sleep, VPSG is essential in case of doubtful episodes to define their epileptic nature or not and therefore direct us toward the most appropriate therapy.

About SDs therapy, even if there is moderate-to-low level of evidence in children with NDDs, parent-based education and behavioral interventions are recommended. Melatonin is the first line pharmacological option at dosages up to 15 mg mainly to regularize nocturnal sleep and reduce the number of awakenings. If ineffective, first generation antihistamines or benzodiazepines (clonazepam) may be considered (9).

In our sample children receiving melatonin did not show better parameters in either SDSC or VPSG, however, this result is not completely reliable as the number of treated individuals was extremely low.

The small sample size also precluded the assessment of a possible influence of the different AEDs.

SDSC questionnaire revealed a high prevalence of SDs, with almost all the sample having borderline or pathological scores, particularly in the subscales concerning initiating and maintaining sleep (DIMS) and the presence of breathing disorders (SBD). SBD was the most compromised sub-scale, reaching pathological values in half of the PKS children. These data differ substantially from those reported by Kostanecka, where the prevalence was much lower (11), most likely due to the higher sensitivity and specificity of the SDSC scale compared to the scale they used in identifying sleep disorders (the ABC analyzes behavioral aspects with only a few generic questions on sleep).

SBD subscale scores were directly correlated to AHI on VPSG revealing that this item is effective in estimating respiratory aspects and their severity. The AI and the AWI did not correlate with the DA, probably because the questions in this item are not well tailored to children with severe disabilities.

These findings support the use of the SDSC questionnaire as a screening tool for SDs in PKS; in first-level centers, it could be essential to identify children to be sent for full VPSG in second and third-level clinics.

Limits: It was not possible to compare the parasomnia-like events of patients with PKS to the then controls, because video of the controls was not available.

## Conclusions

This work contributed to better elucidate SDs in PKS from both a clinical and instrumental point of view.

VPSG revealed a high prevalence of SDs and the presence of pathological sleep macrostructure, with abnormalities in most of the parameters analyzed, especially a reduction in SEI and an increase in the AI and the percentage of N3.

There may be an age-dependent evolution of sleep architecture, with nocturnal awakenings and increased proportion of N1 in younger patients and increased time spent in bed in older patients.

The SDSC questionnaire proved to be a reliable tool for screening SDs in PKS, having significant severity predictivity for breathing disorders. It might then be introduced into clinical practice as a screening tool to identify children to be sent for full VPSG.

These data represent a starting point for the expansion of current knowledge about sleep in PKS, which can be achieved by future more in-depth studies extended to larger cohorts of patients. Finally, as it was not possible to characterize all nocturnal awakenings recorded, further studies will be also necessary to clarify their nature and define any possible influence on sleep macrostructure.

## Data Availability Statement

The raw data supporting the conclusions of this article will be made available by the authors, with the exception of data tracing to single patients.

## Ethics Statement

The studies involving human participants were reviewed and approved by Comitato Etico di Area Vasta Emilia Centro della Regione Emilia-Romagna (CE-AVEC). Written informed consent to participate in this study was provided by the participants' legal guardian/next of kin.

## Author Contributions

VD, DC, and AF conceived the study and developed the study design with FP. MR, VD, SU, AF, and LS acquired the data. MR and AF analyzed the data. AF, LS, GS, MR, and VD wrote the initial draft of the manuscript. All authors contributed to interpretation of the data and critical revision of the manuscript and approved the final version.

## Conflict of Interest

The authors declare that the research was conducted in the absence of any commercial or financial relationships that could be construed as a potential conflict of interest.

## Publisher's Note

All claims expressed in this article are solely those of the authors and do not necessarily represent those of their affiliated organizations, or those of the publisher, the editors and the reviewers. Any product that may be evaluated in this article, or claim that may be made by its manufacturer, is not guaranteed or endorsed by the publisher.
